# Serum PFAS levels in UK firefighters

**DOI:** 10.1038/s41598-026-58059-z

**Published:** 2026-08-03

**Authors:** Anna A. Stec, Thomas Snelgrove, Kathryn Riley, Katarina Handlovicova, Colin C. Everett, Matthew Gittins

**Affiliations:** 1 Centre for Fire and Hazards Sciences, University of Central Lancashire, Preston, PR1 2HE UK; 2 Centre for Biostatistics, School of Health Sciences, Faculty of Biology, Medicine and Health, The University of Manchester, Manchester, M13 9PL UK

**Keywords:** PFAS, PFOA, PFOS, Firefighters, Firefighting foams, Serum, Biomarkers, Environmental sciences, Risk factors

## Abstract

Per- and polyfluoroalkyl substances (PFAS) represent an emerging concern due to their persistence, bioaccumulation, and emerging associations with different cancers and other diseases. Biomonitoring studies worldwide report elevated PFAS serum concentrations in firefighters compared with other occupations and the general population. The aim of this study was to quantify PFAS serum concentration in current and former firefighters and a general population comparison group in the UK. PFAS serum concentrations were analysed in a total sample of 1224 participants, including 942 firefighters from eight Fire and Rescue Services (FRSs) and 282 individuals from the general population. PFAS concentrations varied across tested FRSs, with geometric mean serum levels for some analytes higher than those observed in the general population. These included PFHxS (4.1 times higher), PFOS sum (1.5 times higher) and PFHPS (about 1.6 times higher), compounds that have been historically associated with the use of aqueous film forming firefighting foams. Furthermore, four PFAS (8:2 FTS, FOSA, PFBS, and PFPeS) were detected exclusively in firefighter serum and not in the general population, of which three are commonly detected in firefighter turnout gear. Descriptively, PFAS geometric mean concentrations tended to be higher among older firefighters and those with longer service duration. The general population cohort, drawn from a region which has subsequently been found to have some of the UK’s highest PFAS environmental contamination, showed relatively high baseline serum PFAS levels, suggesting the contribution of high background environmental exposure in this cohort. Reported data highlights the need for PFAS monitoring among firefighters, including evaluation of occupational exposure pathways, with attention to likely sources such as firefighting foam types and formulations; the type of chemicals applied to turnout gear; PPE use, frequency and type of decontamination. Further research should also include wider monitoring of the general population, including evaluation of likely sources and pathways of exposure.

**Supplementary Information:**

The online version contains supplementary material available at 10.1038/s41598-026-58059-z.

## Introduction

Per- and polyfluoroalkyl substances (PFASs) are a wide group of synthetic chemicals. Their strong carbon–fluorine bonds give them very low surface energy, resulting in unique physical and chemical properties, making them useful in a wide range of industrial and consumer applications (e.g. firefighting foams, stain repellents, food-packaging materials, pharmaceuticals, cosmetics, waxes, and adhesives etc.)^1,2^. Carbon–fluorine bonds do not occur in nature, they do not biodegrade, thus, they are highly persistent. Toxicity studies highlight concerns about their long-term effect on the environment and human health^3–6^.

PFAS are not readily metabolised in humans, leading to long biological half-lives. Once absorbed, they bind to serum proteins and accumulate in the liver, kidneys and lungs rather than adipose tissue^7^. Epidemiological studies report adverse health outcomes such as dysfunction of the liver, pancreas, thyroid and immune functions, decreased fertility, elevated low-density lipoprotein and total cholesterol levels, non-Hodgkin’s lymphoma, kidney, testicular and prostate cancers^3,8–10^.

Firefighters’ studies measuring PFAS have shown elevated blood concentrations. While it is recognised that firefighters are occupationally exposed to fire effluents via inhalation, ingestion and/or dermal absorption, for PFAS, the main exposure routes are thought to be inhalation of aerosolised flourosurfactants-based aqueous film-forming (AFFF) firefighting foams, direct or indirect skin contact and hand-to-mouth transfer following use of foams, transfer from (contaminated and off-gassing) PFAS containing and treated personal protective equipment (PPE)^11–17^. Table 1 shows a summary of PFAS compounds identified as one of the main sources of occupational exposure for firefighters. The most extensively reported PFAS include perfluoroalkyl acids (PFAAs), particularly longer-chain compounds such as perfluorooctanoic acid (PFOA), perfluorohexane sulphonate (PFHxS), and perfluorooctane sulphonate (PFOS)^18,19^.

**Table 1:** PFAS analytes identified in different sources.

Identified sources related to PFAS exposure	PFAS analytes detected	References
AFFF (firefighting foams)	PFOS, PFOA, PFHxS, 6:2 FTSA, 6:2 FTAB, 6:2 FtSaAM	^20^
	PFBS, PFHxS, PFHpS, PFOS, PFDS, PFBA, PFPeA, PFHxA, PFHpA, PFOA, PFNA, PFDA, PFUdA, PFDoA, PFTrDA, PFTeDA, 5:3 FTCA, 6:2 FTUCA, 6:2 FTCA, 6:2 FTS, 8:2 FTS, GenX	^21^
Firefighter PPE	PFBA, PFPeA, PFHxA, PFHpA PFOA, PFNA, PFDA, PFUnA, PFDoA, PFBS, PFOS 6:2 FTS, 8:2 FTS	^13^
	PFBA, PFPeA, PFHxA, PFHpA PFOA, PFNA, PFDA, PFUdA, PFDoA, PFTrDA, PFTeDA, PFPrS, PFBS, PFHxS, PFOS, FBSA, N-MeFOSAA	^16^
	8:2 FTOH, 10:2 FTOH, PFBA, PFPeA, PRHxA, PFOA, PFNA, PFDA, PFUdA, PFDoA, PFTrDA, PFTeDA, PFBS, PFOS, FBSA, 6:2 FTSA, 8:2 FTSA	^22^
Fire station dust	6:2 FTS, N-EtFOSAA, 8:2 FTS, PFOA, PFOS, PFHxA, PFNA, PFHpA, PFDA, PFUnDA, PFDoDA, PFTrDA, PFTeDA, PFBA, PFDS, PFPeA, FOSA, N-MeFOSAA, PFHxS, PFBS, PFPeS, PFHpS, 4:2 FtS, PFNS	^17^

The aim of this study was to quantify the PFAS serum concentration in current and former firefighters across United Kingdom Fire and Rescue Services alongside concentrations measured in a sample of the general population.

## Methods

### Ethical approval

Ethical permission for the study was obtained from the Integrated Research Application System for research in health and social care within the UK (IRAS number, IRAS266880) and was conducted in accordance with the ethical principles and guidelines outlined in the Declaration of Helsinki. A memorandum of understanding has been signed with all participating Fire and Rescue Services and informed consent was obtained from all participants.

### Recruitment

**Fig. 1:**
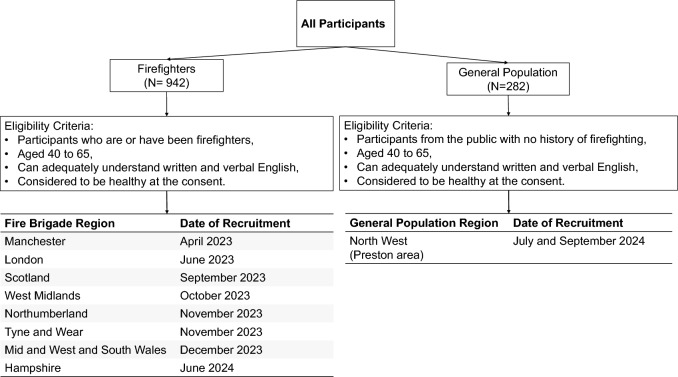
Participant criteria for firefighter cohorts and general population.

There were two distinct cohorts, a firefighter cohort (eight different Fire and Rescue Services, FRSs) and cohort sampled from the general population from around Preston, North West England (Fig. 1).

Firefighters were recruited through the FRSs and Fire Brigades Union. The firefighters recruited were in the age range 40–65 years and included both retired and active firefighters in a variety of roles. The firefighter eligibility criteria for inclusion into the study is shown in Fig. 1.

The general population cohort was recruited from the England’s North West (Preston) region via print advertising (posters/flyers and press releases), email and broadcast media. The participants were included in the study if they had not participated in any firefighting related activities and met the same age requirements as the firefighters.

The total sample size was 1224 participants (942 firefighters and 282 individuals from the general population). This excluded a small number of participants (n = 59) who fell outside the eligible age range.

In total, nine mobile health monitoring clinics were held across England, Scotland and Wales for recruited firefighters and members of the public, the locations can be seen in Fig. 1. All participants were asked to attend a clinic to provide biological samples, have clinical measurements taken and complete a survey. Prior to consent, all participants had the study explained to them in an individual face-to-face appointment and were provided with a participant information leaflet.

### Questionnaire

The questionnaire was set up by using the Research Electronic Data Capture (REDCap) platform, hosted by University of Central Lancashire. Firefighters were asked to complete a questionnaire which detailed basic demographics, workplace demographics, exposure to fire related toxicants, lifestyle information, general health and past or present disease occurrence. The general population were given a questionnaire which excluded questions relating to the fire service and fire exposure.

### Sample collection

Blood samples were collected by a qualified National Health Service (NHS) research nurses using the Sarstedt S-Monovette blood collection system.

### Sample preparation

Following blood collection, all serum sample tubes were stored in an upright position and allowed to clot for 30 min. Then, using a swing-out rotor centrifuge (Camlab DM0412), blood samples were spun for 10 min at 4300 rpm at 20 °C. Obtained serum was aliquoted, using glass pipettes, into sterile 5 mL polypropylene vials, obtained from Elkay Laboratory Products. To ensure sample stability, all serum samples were immediately frozen at − 20 °C and prepared for transportation to the University of Central Lancashire where they were stored at − 80 °C upon arrival, prior to analysis.

### Clinical measurements

During attendance at the health clinics all participants had basic clinical measurements taken. This included weight, height, but also waist circumference, blood pressure, heart rate, oxygen saturation (SpO_2_), and spirometry (reported elsewhere). Body Mass Index (BMI) have been classified according to national UK guidelines, as presented in Table 2^23^.

**Table 2:** Clinical measurements and reference ranges^23^.

Clinical measurement	Device and procedure		Reference range
Weight	Device	Salter Doctor Style Mechanical Scale (Salter, Oldham, UK)	Used for BMI calculation
	Procedure	Weight taken after removing shoes and measured in kilograms	
Height	Device	Stadiometer (Seca 213 Portable Height Measure, Seca, Birmingham, UK)	Used for BMI calculation
	Procedure	Standing height taken with shoes off and measured in centimetres	
Body mass index (BMI) in kg/m^2^	Calculated from weight (kg)/ height (m^2^)		< 18.5—underweight 18.5–24.9—healthy weight 25–29.9—overweight 30 +—obese

### Sample analysis

Details of chemicals and standard compounds used for PFAS analysis have been provided in Table 3 and the Supplementary Information. Serum samples were extracted and analysed using previously established procedures, based on isotope dilution, using a Waters Ultra-Performance ACQUITY H-Class Ultra-high performance liquid chromatography (UHPLC). Coupled to the UHPLC was a XEVO triple quadrupole mass spectrometer (TQMS) equipped with an electrospray ionisation source (ESI). The TQMS was operated in negative ion mode and Mass Lynx 4.10 software was used to process the data and quantify instrumental response. Serum samples (200 µL) were spiked with a PFAS internal standard mixture and subjected to protein precipitation, using 800 µL acetonitrile containing 1% formic acid (v/v). After centrifugation, the supernatant was evaporated under nitrogen and reconstituted in 60 µL H₂O:MeOH (25:75, v/v) containing 2 mM ammonium acetate and performance standard (perfluoro-n-[4-13C4] octanoic acid (50.0 µg/mL ± 2.5 µg/mL) obtained from Wellington Laboratories. Limits of detection (LOD) and quantification (LOQ) were determined according to a signal-to-noise ratio of 3:1 and 10:1, respectively. Full details of the extraction method and analytical settings have been reported in the Supplementary Information.

**Table 3:** Analysed PFAS, their abbreviations and number of carbons (C).

PFAS classification	PFAS analyte name	Cas number	Abbreviation	Number of carbons
Perfluoroalkyl sulphonic acids	Perfluorobutanesulphonic acid	375-73-5	PFBS	C4
	Perfluoropentanesulphonic acid	2706-91-4	PFPeS	C5
	Linear and branched isomers perfluorohexanesulphonic acid	355-46-4	PFHxS	C6
	Perfluoroheptanesulphonic acid	375-92-8	PFHpS	C7
	Sum of linear and branched perfluorooctanesulphonic acid	n/a	∑PFOS	C8
	Perfluorononanesulphonic acid	68259-12-1	PFNS	C9
	Perfluorodecanesulphonic acid	335-77-3	PFDS	C10
Perfluoroalkyl carboxylic acids	Perfluorobutanoic acid	375-22-4	PFBA	C4
	Perfluorohexanoic acid	307-24-4	PFHxA	C6
	Perfluoroheptanoic acid	375-85-9	PFHpA	C7
	Perfluorooctanoic acid	335-67-1	PFOA	C8
	Perfluorononanoic acid	375-95-1	PFNA	C9
	Perfluorodecanoic acid	335-76-2	PFDA	C10
	Perfluoroundecanoic acid	2058-94-8	PFUnDA	C11
	Perfluorododecanoic acid	307-55-1	PFDOA	C12
	Perfluorotridecanoic acid	72629-94-8	PFTrDA	C13
	Perfluorotetradecanoic acid	376-06-7	PFTeDA	C14
Fluorotelomer sulphonates	4:2 Fluorotelomer sulphonic acid	757124-72-4	4:2 FTS	C6
	8:2 Fluorotelomer sulphonic acid	39108-34-4	8:2 FTS	C10
Perfluorinated sulphonamidoacetic acids	Linear and branched isomers N-methyl-perfluorooctanesulphonamido-acetic acid	2355-31-9	N-MeFOSAA	C8
	Linear & branched isomers N-ethyl perfluorooctanesulphonamido- acetic acid	2991-50-6	N-EtFOSAA	C8
Perfluoroethers	Perfluoro(2-((6-chlorohexyl)oxy)ethanesulphonic acid)	756426-58-1	9-Cl-PF3ONS	C6
	11-chloroeicosafluoro-3-oxaundecane-1-sulphonic acid	763051-92-9	11-Cl-PF3ONS	C8
Perfluorinated sulphonamides	Perfluorooctanesulphonamide	754-91-6	FOSA	C8
Perfluoroalkyl ether carboxylic acid	Sodium dodecafluoro-3H-4,8-dioxanonanoate	2250081-67-3	NaDONA	C7
	Hexafluoropropylene oxide-dimer acid	13252-13-6	HFPO-DA	C6

### Data analysis

Data analyses presented in this manuscript were restricted to descriptive analyses of PFAS serum concentrations. Adjusted multivariable statistical models examining occupational, demographic, lifestyle, and other determinants of PFAS exposure will be reported in a separate manuscript.

Data cleansing and grouping was undertaken both manually and using automated methods to ensure that the study results are accurate and reproducible. Additional checks for data accuracy included graphical analysis of result distributions, cross-tabulations and production of summary statistics.

All statistical analyses were performed using RStudio (R version 4.5.1). Analysis focused on summarising concentrations of different PFAS analytes across the two cohorts (firefighters and the general population). For each PFAS analyte, detection frequency (DF, %) was calculated as the percentage of samples with concentrations above the analytical limit of detection (LOD).

To characterise the PFAS concentrations’ variability, the minimum and maximum values were calculated along with the range (defined as the maximum minus the minimum), and the maximum-to-minimum ratio. In addition to descriptive statistics, geometric means (GM) with 95% confidence intervals (CI), along with percentiles were reported for analytes for any samples having detection frequency ≥ 60%. Relative variability was assessed using the coefficient of variation (CV).

Measurements below the LOD were imputed using LOD/√2^24^. These were included in calculations of minimum, maximum, range, ratio and percentiles values. Samples below the LOD were excluded from calculations of geometric mean, confidence intervals and coefficient of variation which were based solely on detectable concentrations.

In addition, results are presented as ∑_NASEM_PFAS, in accordance with recently published following recently the United States’ National Academies of Sciences, Engineering, and Medicine (NASEM) guidance on PFAS exposure, testing, and clinical follow-up. One of their recommendations directs clinicians to use the sum of seven PFAS (N-MeFOSAA, PFHxS, ∑PFOA, PFDA, PFUnDA, ∑ PFOS, and PFNA) detected in serum to inform clinical care of exposed patients. NASEM’s proposed risk thresholds for total serum PFAS (∑_NASEM_PFAS) are: < 2 ng/mL—adverse health effects not expected; 2 to < 20 ng/mL—potential for adverse health effects, especially in sensitive populations; ≥ 20 ng/mL—increased risk of adverse health effects^25^.

## Results

Results are presented separately for the general population and combined for all tested FRSs. The total sample size was 1224 participants: 942 firefighters and 282 individuals from the general population. Eight FRSs were included in the health monitoring, covering three of the UK nations, England, Scotland and Wales. The majority of the control cohort participants, 90.4%, were employed in Lancashire (North West) region. Demographic and occupational information are summarised in Tables 4 and 5, respectively.

**Table 4:** Demographic, body mass index and smoking characteristics of all participants (N = 1224) in two cohorts. Values are presented as percentages with the total numbers (N) in parentheses.

Characteristics	General population % (N)	FRSs % (N)
**Sex**		
Male	89.0 (251)	92.3 (869)
Female	11.0 (31)	7.7 (73)
**Ethnicity**		
White-British	89.0 (251)	90.6 (853)
Non-White	5.3 (15)	2.9 (27)
White-Other	5.7 (16)	6.6 (62)
**Age (mean (years ± SD)**	51.33 ± 7.34	49.83 ± 6.26
40–44	23.4 (66)	24.5 (231)
45–49	23.8 (67)	25.4 (239)
50–54	15.6 (44)	26.6 (251)
55–59	18.1 (51)	14.9 (140)
60–65	19.1 (54)	8.6 (81)
**Body mass index**		
Healthy	27.7 (78)	14.5 (137)
Overweight	46.1 (130)	53.9 (508)
Obese	25.5 (72)	31.5 (297)
Underweight	0.8 (2)	0.0 (0)
**Smoking (%, (N))**		
Never	73.0 (206)	64.6 (609)
Ever	16.7 (47)	25.5 (240)
Current	8.9 (25)	9.4 (89)
Unknown	1.4 (4)	0.4 (4)

**Table 5:** Firefighters occupational characteristics; N = total number.

	FRSs (N = 942)
**Years in service** Mean ± SD	23.2 ± 6.5
**Occupational status (%, (N))**	
Firefighter	34.1 (321)
Instructor	0.3 (3)
Watch manager	19.3 (182)
Crew manager	13.1 (123)
Station manager	6.3 (59)
Group manager	2.9 (27)
Area manager	0.7 (7)
Principal manager	0.2 (2)
Other	1.5 (14)
Retired (no longer active)	21.7 (204)
**Number of significant fires attended (%, (N)**)	
No attendance/other	3.4 (32)
1–3 per month	72.3 (681)
1–2 per week	14 (132)
3–5 per week	6.8 (64)
> 5 per week	3.5 (33)

The distribution of ages between sexes is similar in the control cohort and all FRSs. The firefighter sample population had a lower percentage of female staff compared with national fire-service figures (2023: England 19.4%, Wales 10%, Scotland 14.4%), but a similar male/female ratio was present in the general population cohort^26–28^.

The mean age of the general population participants tested was 51.3 ± 7.3 years, which is similar to the firefighter population (49.8 ± 6.3 years). The firefighter cohort had a higher prevalence of BMI results classified as overweight or obese (85.4%), compared with the general population cohort (71.6%). For smoking habits, variance is linked to the tested region. For current smokers, overall results were comparable between the general population (8.9%) and all FRSs (9.4%).

On average, 34% of participants in the firefighter cohort reported an occupational status as a firefighter. Among the remaining participants, over 40% held supervisory roles (watch manager level and above). In total, 22% of the cohort had retired shortly (within the last 3 years) before the sample collection. More than 65% of all recruited firefighters had spent over 20 years in the UK Fire and Rescue Service (with 30% serving 20–25 years, 23% serving 25–30 years, and 15% serving 30–35 years).

The mean of the firefighters’ years in service was 23.2 ± 6.5 years. Depending on the subgroup, 65–83% of tested firefighter participants attended 1–3 significant fires per month.

Table 6 presents the serum PFAS geometric mean concentrations with 95% confidence interval (CI) and coefficients of variation (CV), together with minimum and maximum values, ranges, and maximum-to-minimum ratios for the two study cohorts, with male and female participants combined within each cohort.

**Table 6:** Serum PFAS concentrations (ng/mL) presented as geometric means (GM) with 95% confidence intervals (CI), percentiles and other descriptive statistics for firefighters and the general population; *FRSs* Fire and Rescue Services, Range is defined as the maximum minus the minimum value, Ratio is defined as maximum value-to-minimum value; *DF* detection frequency, *CV* Coefficient of variation.

Analyte	Group	DF (%)	Maximum value (ng/mL)	Range (ng/mL)	Ratio	Geometric mean (95% CI) (ng/mL)	CV
PFOA	General population	100	7.07	7.07	4168.69	1.46 (1.37, 1.54)	52.55
	FRSs	100	12.33	12.17	77.79	1.14 (1.10, 1.18)	61.23
∑PFOS	General population	100	19.86	19.85	4135.69	2.41 (2.24, 2.60)	80.44
	FRSs	100	13.69	13.45	57.89	2.73 (2.63, 2.82)	55.01
PFHxS	General population	100	4.58	4.58	1236.90	0.77 (0.71, 0.83)	65.01
	FRSs	100	24.99	24.94	537.33	1.43 (1.36, 1.51)	93.76
PFNA	General population	100	1.57	1.56	418.39	0.26 (0.25, 0.28)	66.33
	FRSs	100	2.18	2.18	581.43	0.28 (0.28, 0.29)	46.77
PFDA	General population	92	0.69	0.68	142.88	0.11 (0.10, 0.12)	70.98
	FRSs	95	0.98	0.97	203.28	0.12 (0.11, 0.12)	54.66
PFHPS	General population	83	0.53	0.52	114.92	0.11 (0.11, 0.12)	55.29
	FRSs	89	0.70	0.70	152.07	0.14 (0.14, 0.14)	52.30
PFUNDA	General population	69	0.46	0.46	363.03	0.09 (0.09, 0.10)	59.49
	FRSs	76	0.42	0.42	330.64	0.09 (0.09, 0.09)	43.96
PFBA	General population	15	0.48	0.47	52.10	0.08 (0.07, 0.09)	80.66
	FRSs	20	0.28	0.27	30.54	0.07 (0.07, 0.08)	41.20
N-MeFOSAA	General population	8	1.89	1.88	204.72	0.20 (0.13, 0.31)	149.28
	FRSs	10	0.58	0.57	62.43	0.12 (0.10, 0.13)	75.26
PFHPA	General population	3	0.45	0.44	53.41	0.15 (0.09, 0.26)	70.61
	FRSs	4	0.45	0.44	53.23	0.13 (0.12, 0.15)	56.63
PFDoA	General population	4	0.11	0.10	67.84	0.07 (0.05, 0.08)	33.68
	FRSs	3	0.26	0.26	164.67	0.06 (0.06, 0.07)	57.10
PFTrDA	General population	4	0.08	0.08	21.25	0.06 (0.05, 0.07)	19.20
	FRSs	2	0.12	0.12	30.11	0.06 (0.06, 0.07)	23.97
PFTeDA	General population	1	0.07	0.06	8.73	0.07 (0.05, 0.09)	10.77
	FRSs	< 1.0	0.10	0.09	11.89	0.08 (0.06, 0.10)	19.95
PFPeS	FRSs	16	0.19	0.18	40.03	0.08 (0.07, 0.08)	34.65
8:2 FTS	FRSs	< 1.0	0.09	0.08	95.40	0.07 (0.03, 0.13)	25.69
PFBS	FRSs	2	4.97	4.97	2342.92	0.11 (0.05, 0.24)	263.61
FOSA	FRSs	< 1.0	0.34	0.33	20.61	0.17 (0.05, 0.58)	67.84

Of the 26 PFAS analytes measured, 8 of them (11-Cl-PF3OUdS (C11), PFDS (C10), NaDONA (C9), PFNS (C9), 9-Cl-PFOUdS (C8), HFPO-DA (C6), PFHxA (C6), and 4:2 FTS (C6)) were not detected in either cohort. N-EtFOSAA was detected in only one sample in each cohort. The other 17 PFAS analytes were found and quantified across both cohorts and are presented in Table 3.

Several analytes were detected only in firefighters and not in the general population. These include: 8:2 FTS (C10), FOSA (C8), PFPeS (C5), and PFBS (C4) suggesting potential occupational exposure pathways. However, their low detection frequencies and high variability limit definitive interpretation. In contrast, PFHxS was consistently detected in both cohorts, with geometric mean concentrations nearly twice as high in firefighters compared with the general population (1.4 vs. 0.8 ng/mL).

The longer-chain PFAAs (C6 to C11): perfluoroalkyl sulphonic acids and perfluoroalkyl carboxylic acids represent the main group of identified PFAS across both studied cohorts, with DFs > 60%. These analytes are: PFUnDA (C11), PFDA (C10), PFNA (C9), PFOA (C8), ∑PFOS (C8, sum of liner and branched), PFHpS (C7), PFHxS (C6) where ∑PFOS, PFHxS and PFOA were detected in all samples.

PFOA and ∑PFOS showed higher geometric mean concentrations in the general population compared with FRSs. For a few analytes, ranges and geometric mean concentrations were higher among FRSs compared with the general population. PFHxS. PFHxS showed a pronounced difference, with a geometric mean nearly twice as high in FRSs (1.43 ng/mL) compared with the general population (0.77 ng/mL). Similarly, ∑PFOS (sum of linear and branched isomers)) concentrations were slightly higher in FRSs (2.73 ng/mL) relative to the general population (2.41 ng/mL). PFOA geometric mean concentrations, on the other hand, were slightly lower in FRSs than in the general population.

Variability in PFAS concentrations differed between cohorts. The general population exhibited much larger maximum-to-minimum ratios for several analytes, including PFOA and ∑PFOS, with ratios exceeding 4000. These very high ratios were driven primarily by very low minimum concentrations, often at or near the analytical limit of detection, combined with a small number of individuals exhibiting comparatively high concentrations. In contrast, ratios observed among firefighters were lower (below 100), indicating a more homogeneous exposure distribution within this occupational cohort.

For both cohorts, coefficients of variation were generally low to moderate across most PFAS analytes. However, the general population often exhibited slightly higher CVs, indicating greater inter-individual variability in exposure which in some cases may also be influenced by a small number of individuals with higher PFAS concentrations. In contrast, CVs in the tested FRSs were more consistently low to moderate, indicating more similar PFAS concentrations among firefighters. This pattern suggests shared exposure sources or comparable exposure intensity and is consistent with more homogeneous exposure profiles typically associated with occupational settings.

Table 7 presents the geometric means and 95% CI for these PFAS for both tested cohorts, along with several other characteristics. Based on the NASEM guidelines, results show that more than 90% of participants (91% general population members and 93% of tested firefighters) fell within the intermediate-exposure category (2 to < 20 ng/mL), with only around 1% (1.4% general population members and 0.5% firefighters) below 2 ng/mL. For values exceeding the ≥ 20 ng/mL threshold, associated with increased risk of adverse health effects, 4.3% of the general population and nearly 2% of firefighters fell into this category.

**Table 7:** Total PFAS serum concentrations (∑_NASEM_PFAS, ng/mL), according to the United States’ National Academies of Sciences, Engineering, and Medicine, presented as geometric means (GM) with 95% confidence intervals (CI) and other descriptive statistics for firefighters and the general population; *DF* detection frequency, *CV* Coefficient of variation; Range is defined as the maximum minus the minimum value; Ratio is defined as maximum value-to-minimum value.

Characteristics	Cohort	Maximum value (ng/mL)	Range (ng/mL)	Ratio	Geometric mean (95% CI) (ng/mL)	CV
**Sex**						
Male	General population	28.56	28.53	976.13	5.29 (4.91, 5.69)	55.34
	FRSs	42.17	41.32	49.59	6.29 (6.08, 6.50)	52.36
Female	General population	28.84	27.63	23.70	4.54 (3.48, 5.92)	99.05
	FRSs	19.53	18.92	31.91	4.03 (3.51, 4.63)	62.28
**Ethnicity**						
White-British	General population	28.84	28.81	985.79	5.27 (4.88, 5.69)	60.64
	FRSs	42.17	41.55	68.90	5.96 (5.75, 6.17)	53.90
Non-White	General population	17.31	16.10	14.22	4.74 (3.34, 6.72)	72.01
	FRSs	22.77	19.19	6.36	7.72 (6.61, 9.01)	46.03
White-Other	General population	15.24	13.62	9.41	4.63 (3.43, 6.26)	61.10
	FRSs	27.42	25.12	11.94	7.16 (6.31, 8.12)	51.62
**Age**						
40–44	General population	9.82	9.17	15.03	4.28 (3.82, 4.78)	39.12
	FRSs	27.42	26.57	32.25	5.47 (5.09, 5.87)	56.71
45–49	General population	10.37	10.34	354.28	4.51 (3.75, 5.43)	40.23
	FRSs	17.37	15.73	10.56	5.53 (5.22, 5.86)	45.31
50–54	General population	28.56	26.66	15.01	6.05 (5.06, 7.25)	72.10
	FRSs	23.27	22.65	38.02	6.62 (6.22, 7.06)	48.74
55–59	General population	22.50	20.88	13.88	5.52 (4.77, 6.38)	63.82
	FRSs	22.77	21.81	23.72	6.88 (6.30, 7.51)	49.35
60–65	General population	28.84	27.82	28.29	6.58 (5.69, 7.61)	56.75
	FRSs	42.17	40.79	30.74	6.72 (5.93, 7.62)	68.40
Body mass index						
Healthy	General population	28.84	28.19	44.14	5.01 (4.42, 5.68)	67.96
	FRSs	23.27	22.65	38.02	5.71 (5.17, 6.31)	59.83
Overweight	General population	28.56	26.77	15.91	5.74 (5.28, 6.25)	61.18
	FRSs	19.53	18.68	22.97	6.05 (5.79, 6.33)	48.14
Obese	General population	12.71	12.68	434.47	4.50 (3.71, 5.45)	47.40
	FRSs	42.17	40.79	30.74	6.28 (5.92, 6.66)	59.03
**Occupational status**						
Active firefighter		42.17	41.55	68.90	5.90 (5.69, 6.12)	54.42
Retired		21.33	20.37	22.22	6.74 (6.25, 7.27)	50.21
1–3 year		42.17	41.55	68.90	6.10 (5.86, 6.35)	53.00
1–2 per week		23.27	21.89	16.96	6.01 (5.51, 6.56)	51.46
3–5 per week		27.42	25.57	14.80	5.95 (5.26, 6.73)	61.06
> 5 per week		21.33	19.89	14.75	6.02 (4.90, 7.39)	60.62

Analysing demographic characteristics, presented in Table 7, ∑_NASEM_PFAS geometric mean concentrations were found to be 19% higher in male firefighters (6.3 ng/mL) compared to the general population (5.3 ng/mL). This is driven primarily by sulphonic PFAS, particularly PFHxS (+ 86%), ∑PFOS (+ 13%), and PFHpS (+ 25%), all of which were consistently higher in firefighters. While PFHxS is not used in firefighting foams, it is a breakdown product of PFOS/PFOA which are commonly found in both firefighting gear and firefighting foams. Perfluorinated carboxylic acids showed only modest differences in male firefighters compared with the general population, with both PFNA and PFDA 7%, higher, while PFOA and PFUnDA were approximately 22% and 4% lower, respectively.

Across all age groups, FRSs have higher ∑_NASEM_PFAS geometric means in every age group when compared to the general population. The differences are particularly seen at the younger age 40–44 and 45–40 years old. ∑_NASEM_PFAS concentrations varied also across demographic groups, with males showing higher values than females in both firefighters and the general population. Geometric mean concentrations were higher in male firefighters than females (6.3 versus 4.0 ng/mL). A similar pattern was observed in the general population, although the difference between males and females was smaller (5.3 versus 4.5 ng/mL).

While non-white firefighters had approximately 1.5 times higher geometric mean for the ∑_NASEM_PFAS (7.7 ng/mL) than white-British individuals (6.0 ng/mL), this trend is not observed across the general population (4.7 vs 5.2 ng/mL). Looking at participants’ ages, an accumulative pattern in ∑_NASEM_PFAS is observed, with concentrations increasing with age in both cohorts. Overall, firefighters showed higher geometric mean concentrations than the general population in most age groups, particularly in younger age categories.

Higher ∑_NASEM_PFAS geometric mean concentrations were observed among overweight and obese firefighter participants compared to the general population. While higher BMI may be associated with higher PFAS concentrations in blood, other factors, such as firefighters’ occupational exposures, may also contribute to the observed differences. Occupational variables among firefighters showed that retired personnel (with longer years of service) had higher ∑_NASEM_PFAS geometric means than active firefighters (6.7 versus 5.9 ng/mL; a 14% increase).

Across most FRSs, geometric mean PFAS concentrations together with the overlapping confidence intervals were comparable across different firefighters’ frequencies of fire attendance. Lack of association with PFAS may be explained by the infrequent use of PFAS-containing firefighting foams which are typically used only during specific types of fire incidents (e.g. fuel storage tank or industrial chemical warehouse fires).

## Discussion

Both the general population and firefighters will be exposed to PFAS through environmental sources. In the UK, a report for the Environment Agency (EA) has identified more than 10,000 high risk sites contaminated with PFAS, with an estimated remediation cost of between £31–121 billion^29^. The EA data analysis by Wildlife & Countryside Link and the Rivers Trust found that 77% (81 out of 105) of English river sites where PFAS had been detected contained levels that do not meet the tougher proposed EU environmental quality standard (where the sum of PFAS, weighted as PFOA-equivalents, exceeds 4.4 ng/L limit), with some breaching it by 10 or 20 times^30^.

The general population sample was drawn from residents of Lancashire, the North West region of England, which include the River Mersey catchment, a region where recent studies report PFAS export among the highest globally^31^. Additionally, several sites in the region may contribute to environmental PFAS exposure. Angus Fire, Bentham (on the border between Lancashire and Yorkshire), produced PFAS-containing firefighting foams until 2024. The area has been shown to have very high PFAS concentrations in groundwater. Environmental investigations in 2008 at the Angus Fire site revealed total PFAS levels of 1,199,000 ng/L in groundwater samples, including 36,100 ng/L of PFOS. This exceeds the UK environmental quality standard for PFOS by more than 55,000-fold^32,33^. Environmental monitoring at AGC Chemicals Europe site, a Lancashire-based manufacturer of PFAS-related fluoropolymers, has also detected effluent containing more than 700 different PFAS compounds^34^.

While firefighting practice in the UK primarily relies on water as the standard suppression agent for most fire types, firefighting foams are used more selectively for specific high-risk scenarios, particularly Class B fuel fires and certain industrial or aviation-related incidents. Obtained PFAS data support and show that firefighter exposures vary across geographically and demographically diverse FRSs. Additionally, these differences may be linked not only to the background variations, but also to the type and use of firefighting gear or AFFF firefighting foams, as well as differences in firefighters working conditions and their associated exposures (type, duration, pathway, etc.)^35–37^.

Four PFAS analytes (8:2 FTS (C10), FOSA (C8), PFBS (C4), and PFPeS (C5)) were detected exclusively in the firefighter cohorts. Of these: 8:2 FTS (C10), and PFBS (C4) have been widely detected in firefighter turnout gear textiles (jacket and pants) in US and Canadian studies^22^^,^^38^.

Changes in national statutory regulations regarding the use and application of PFAS chemicals, and the timing of their implementation by individual FRSs, may provide some explanation for the observed differences in PFAS geometric mean values. For example, Queensland, Australia, was one of the first jurisdictions to ban PFAS use in firefighting foam in 2016. In the UK, the use of firefighting foams containing C8-PFAS: PFOA and PFOS is scheduled to be banned from July 2025 and January 2026, respectively. However, C6-based PFAS foams (Table 3) are still legally supplied and used at present^39,40^. A survey, conducted by the National Fire Chiefs Council revealed that many FRSs have transitioned from C8-based foams to C6-based foams following plans for the PFAS ban. Several FRSs reported that they are either in the process of disposing of C-6 based foams and replacing them with fluorine-free alternatives or plan to do so in the future^41^. Additionally, FRSs region-specific guidance related to contamination and decontamination strategies implemented by each FRSs may further influence both the levels and types of PFAS analytes detected.

Local FRSs risk assessments also contribute to variability in PFAS exposure and detection. FRSs serving areas with high-risk facilities, such as petrochemical storage sites, are more likely to stock PFAS-containing foams to ensure preparedness for potential large-scale incidents. As in the Fire Industry Association guidance note, older extinguishers or suppression systems that historically contained C8 PFAS may remain non-compliant even after being refilled with C6 foam if residual legacy PFAS or their breakdown products persist above permitted threshold. As a result, firefighters may still be exposed to a mix of C8 and C6 PFAS. This may explain why certain PFAS such as PFOA, PFAS, PFHxS are higher in firefighter serum (while PFHxS is not actively used in contemporary firefighting foams, it can arise as a breakdown product of PFOS and PFOA)^42,43^.

### Limitations

Though the firefighter cohort represents FRSs from across the UK, the results presented here are descriptive statistics of an observational, cross-sectional study of self-selected participants. Results presented here are unadjusted for key confounding variables (such as age, smoking status, socio-economic factors etc.). Therefore, the findings should be interpreted with caution until further analyses examining potential occupational associations among firefighters, with appropriate adjustment for confounders, can be performed. Uncertainty is present when interpreting exposure patterns, especially in FRSs where higher PFAS concentrations were observed since these may reflect differences in the deployment of AFFF foams, the types of foams stored historically, or how frequently they were used in real incidents or training scenarios. A similar case applies to PPE, where PFAS exposure patterns and concentrations are further influenced by the types of gear used, the frequency and methods of replacement, the effectiveness and regularity of decontamination procedures, and variations in individual firefighter practices. Furthermore, information on PFAS content in AFFF (firefighting foams) and PPE used in the UK is limited because formulations used during manufacturing are often proprietary and not publicly disclosed. Collectively, these factors make it challenging to separate occupational exposure pathways and to compare PFAS body burdens across different FRSs.

Additionally, PFAS serum concentrations, particularly for persistent compounds such as PFOS, PFHxS and PFOA, are influenced by background environmental exposures and can vary regionally, therefore, matching control participants to each firefighter sampling site would have provided a more direct comparison. This was not feasible within the present study due to limited resources, and the difficulty of recruiting demographically comparable participants across multiple regions. Further to this, the absence of national-level PFAS monitoring in UK drinking water limits the ability to contextualise regional environmental exposure, making it difficult to determine how background contamination may differ across sites. Instead, the general-population sample was drawn locally from North West England, an area known to have high PFAS levels^31–34^. Even in this case, measured geometric mean values in four FRSs were still higher. Future studies would benefit from regionally matched controls and improved environmental monitoring to better isolate occupational exposure from background variation.

## Conclusions

This study provides the first national overview of PFAS exposure within the UK FRSs. Total NASEM PFAS geometric mean concentrations increased with age and years of service and appear to remain higher among retired personnel, consistent with the cumulative bioaccumulation of long-lived PFAS.

The PFAS levels observed in firefighters were driven primarily by the sulphonic acids, PFHxS and total PFOS, compounds historically associated with AFFF use. Three out of five PFAS analyte that were commonly detected in firefighting gear fabrics and textiles were detected exclusively in firefighter serum and not in the general population, indicating potential differences in exposure sources that may be occupational.

These findings highlight the need for continued PFAS monitoring in the general population, including evaluation of likely sources and pathways of exposure. Whiles appropriately accounting for background exposures, further research should also include occupational exposure pathways among firefighters, with attention to firefighting foam types and formulations, type and chemical composition of turnout gear, PPE use, age and frequency/type of decontamination.

## Supplementary Information

Supplementary Information.

## Data Availability

The datasets generated and/or analysed during the current study are available from the corresponding author on reasonable request.
