# Supplementary material for: Serum PFAS levels in UK firefighters
Source: Sci Rep. 2026 Aug 3;16:23771. doi: 10.1038/s41598-026-58059-z (PMC13434400; doi:10.1038/s41598-026-58059-z)
Supplement: Supplementary file 1 [file 41598_2026_58059_MOESM1_ESM.docx]

**Supplementary Information**

Serum PFAS Levels in UK Firefighters

Thomas Snelgrove^1^, Kathryn Riley^1^, Katarina Handlovicova^1^, Colin Everett^2^, Matt Gittins^2^, Anna A Stec*,

^1^Centre for Fire and Hazards Sciences, University of Central Lancashire, Preston, PR1 2HE, UK

^2^School of Health Sciences, Faculty of Biology, Medicine and Health, The University of Manchester, Manchester M13 9PL, UK

Corresponding author: [aastec@uclan.ac.uk](mailto:aastec@uclan.ac.uk)

**Details of chemicals, standard compounds, sample preparation, and analytical methods used for PFAS analysis**

**Reagents and materials**

All laboratory equipment and reagents used during the sampling and extraction procedure were evaluated for possible PFAS contamination; this included monovettes, eppendorfs, solvents and other used consumables. Any consumables or reagents found to contain PFAS were replaced and retested.

UHPLC grade solvents methanol (≥99.8%) and water (≥99.8%) were obtained from VWR International. LC grade ammonia acetate was obtained from MERCK. Acetonitrile and isopropyl alcohol (HPLC grade ≥99.8%) and formic acid (≥99%) was obtained from VWR.

2ml square-well collection plate and 96-well Square Plug Pre-slit Cap mats were obtained from Waters. LC columns ACQUITY Premier HSS T3 Column (1.8 µm, 2.1 x 100 mm) and PFAS isolator column (2.1 mm X 50 mm) was obtained from Waters Corporation. Foetal bovine serum was utilised as a PFAS free matched matrix and was obtained from MERCK. Stuart sample concentrator was obtained from Peak scientific. A mini centrifuge (Camlab D1008) and clinical bench-top centrifuge (Camlab DM0412) were both obtained from Camlab. White labelled 7.5ml Serum (92mm x 15mm) S-Monovette obtained from Sarstedt.

**PFAS standards**

Thirty PFAS analytes and twenty internal standards were evaluated in this study. PFAC-30 standard solution (1.0 µg/mL ±0.1 µg/mL) in methanol/isopropanol (6%) water (<1%) was utilised as calibration standards obtained from Wellington laboratories presented in Table S1. MPFAC-24ES internal standard solution (1.0 µg/mL ±0.1 µg/mL) in methanol/isopropanol (2%) water (<1%) and M3HFPO-DA internal standard solution (50.0 µg/mL ± 2.5 µg/mL) in methanol were used in combination to create a mixture of twenty internal standards and were both obtained from Wellington Laboratories and are presented in Table S2. A single injection standard Perfluoro-n-(4-13C4) octanoic acid (M4-PFOA) (50.0 µg/mL ± 2.5 µg/mL) in methanol obtained from Wellington Laboratories was used to monitor instrument response and is also shown in Table S2.

**Table S1**: PFAS calibration analytes obtained from PFAC30 solution.

| **PFAS** | | |
| --- | --- | --- |
| **PFAS classification** | **Target PFAS Compounds** | **Abbreviations** |
| **Perfluorinated carboxylic acids** | Perfluorobutanoic acid | PFBA |
|  | Perfluoropentanoic acid | PFPeA |
|  | Perfluorohexanoic acid | PFHxA |
|  | Perfluoroheptanoic acid | PFHpA |
|  | Perfluorooctanoic acid | PFOA |
|  | Perfluorononanoic acid | PFNA |
|  | Perfluorodecanoic acid | PFDA |
|  | Perfluoroundecanoic acid | PFUnDA |
|  | Perfluorododecanoic acid | PFDoDA |
|  | Perfluorotridecanoic acid | PFTrDA |
|  | Perfluorotetradecanoic acid | PFTeDA |
| **Perfluorinated sulfonic acids** | Perfluorobutanesulphonic acid | PFBS |
|  | Perfluoropentanesulphonic acid | PFPeS |
|  | linear & branched isomers Perfluorohexanesulphonic acid | PFHxS |
|  | Perfluoroheptanesulphonic acid | PFHpS |
|  | linear & branched isomers Perfluorooctanesulphonic acid | PFOS |
|  | Perfluorononanesulphonic acid | PFNS |
|  | Perfluorodecanesulphonic Acid | PFDS |
| **Perfluorinated sulphonamides** | Perfluorobutanesulphonic acid | PFBS |
|  | Perfluorohexanesulphonamide | FHxSA |
|  | Perfluorooctanesulphonamide | FOSA |
| **Perfluorinated sulphonamidoacetic acids** | linear & branched isomers  N-Methyl perfluorooctanesulphonamidoacetic acid | N-MeFOSAA |
|  | linear & branched isomers  N-ethyl perfluorooctanesulphonamidoacetic acid | N-EtFOSAA |
| **Fluorotelomer sulfonates** | 4:2 Fluorotelomer Sulphonic Acid | 4:2FTS |
|  | 6:2 Fluorotelomer Sulphonic Acid | 6:2FTS |
|  | 8:2 Fluorotelomer Sulphonic Acid | 8:2FTS |
| **Perfluoroethers** | Sodium dodecafluoro-3H-4,8-dioxanonanoate | NaDONA |
|  | Perfluoro(2-((6-chlorohexyl)oxy)ethanesulphonic acid) | 9Cl-PF3ONS |
|  | 11-chloroeicosafluoro-3-oxaundecane-1-sulfonic acid | 11Cl-PF3OUdS |
| **Perfluoroalkyl ether carboxylic acid** | Hexafluoropropylene oxide-dimer acid | HFPO-DA |

**Table S2**: PPFAS internal standards obtained from MPFAC-24ES - M3HFPO-DA and PFAS injection standard (M4-PFOA)

| **PFAS** | | |
| --- | --- | --- |
| **PFAS classification** | **Target internal standard PFAS Compounds** | **Abbreviations** |
| **Perfluorinated carboxylic acids** | Perfluoro-n-(13C4)butanoic acid | MPFBA |
|  | Perfluoro-n-(13C5)pentanoic acid | M5PFPeA |
|  | Perfluoro-n-(1,2,3,4,6-13C5)hexanoic acid | M5PFHxA |
|  | Perfluoro-n-(1,2,3,4-13C4)heptanoic acid | M4PFHpA |
|  | Perfluoro-n-(13C8)octanoic acid | M8PFOA |
|  | Perfluoro-n-(13C9)nonanoic acid | M9PFNA |
|  | Perfluoro-n-(1,2,3,4,5,6-13C6)decanoic acid | M6PFDA |
|  | Perfluoro-n-(1,2,3,4,5,6, 7-13C7)undecanoic acid | M7PFUndA |
|  | Perfluoro-n-( 1,2-13C2)dodecanoic acid | MPFDoDA |
|  | Perfluoro-n-( 1,2-13C2)tetradecanoic acid | M2PFTeDA |
|  | Perfluoro-n-[4-13C4] octanoic acid | M4PFOA |
| **Perfluorinated sulfonic acids** | Sodium perfluoro-1-(2,3,4-13C3)butanesulphonate | M3PFBS |
|  | Sodium perfluoro-1-(1,2,3-13C3)hexanesulphonate | M3PFHxS |
|  | Sodium perfluoro-1-(13C8)octanesulphonate | M8PFOS |
| **Perfluorinated sulphonamides** | Perfluoro-1-( 13C8)octanesulphonamide | M8FOSA |
| **Perfluorinated sulphonamidoacetic acids** | N-methyl-d3-perfluoro-1-octanesulfonamidoacetic acid | D3-N-MeFOSAA |
|  | N-ethyl-d5-perfluoro-1-octanesulfonamidoacetic acid | D5-N-EtFOSAA |
| **Fluorotelomer sulfonates** | Sodium 1H,1H,2H,2H-perfluoro(1,2-13C2)hexanesulphonate | M2-4:2FTS |
|  | Sodium 1H,1H,2H,2H-perfluoro(1,2-13C2)octanesulphonate | M2-6:2FTS |
|  | Sodium 1H,1H,2H,2H-perfluoro(1,2-13C2)decanesulphonate | M2-8:2FTS |
| Perfluoroalkyl ether carboxylic acid | 2,3,3,3-Tetrafluoro-2-(1,1,2,2,3,3,3-heptafluoropropoxy)-13C3-propanoic acid | M3-HFPO-DA |

**Standard solution preparation**

Commercial PFAC-30 standard solution (1000 ng mL⁻¹), containing 30 PFAS analytes, was transferred from its original amber glass ampoule to a screw-cap amber glass vial with a PTFE liner. The stock solution was subsequently diluted in methanol to prepare 100 ng mL⁻¹ and 10 ng mL⁻¹ working solutions. The 1000, 100, and 10 ng mL⁻¹ PFAC-30 solutions were used for spiking foetal bovine serum (FBS) to prepare calibration standards and quality control (QC) samples. An internal standard (IS) stock solution was prepared from the PFAC-24ESI mixture (1000 ng mL⁻¹) and M3HFPO-DA (50.0 µg/mL) which were transferred from amber glass ampoules to screw-cap amber glass vials. These were mixed and diluted in methanol to obtain a combined IS working solution of 100 ng mL⁻¹.

**Protein Precipitation (PPT)**

Foetal bovine serum (200 µL) was fortified with PFAS standards at the required concentrations and spiked with 10 µL of an internal-standard mixture (100 ng mL⁻¹). ⁻¹). Acetonitrile containing 1% formic acid (800 µL) was added, and samples were vortex-mixed for approximately 30. Samples were agitated at 500 rpm for 30 min to ensure complete protein precipitation and centrifuged for 5 min at 7000 rpm. The supernatant was transferred to a glass vial and evaporated to dryness under nitrogen at 60 °C. Samples were reconstituted in 60 µL of 25:75 H₂O:MeOH (v/v) containing 2 mM ammonium acetate and M4-PFOA recovery standard (c = 10 ng mL⁻¹). The reconstituted extracts were then transferred to a 2 mL 96-well plate and placed into the UHPLC sample manager for analysis.

**UHPLC-MS/MS analysis**

Analytical instrumentation included a Waters Ultra-Performance ACQUITY H-Class Ultra-high performance liquid chromatography (UHPLC). The UHPLC conditions shown in Table S3. Coupled to the UHPLC was a XEVO triple quadrupole mass spectrometer (TQMS) equipped with an electrospray ionisation source (ESI). The TQMS was operated in negative mode and Mass lynx 4.10 software was used to process the data and quantify instrumental response. The MS parameters for acquisition are displayed in Table S4.

**Multiple reaction monitoring**

The multiple reaction monitoring (MRM) was used for quantifying PFAS analytes and internal standards; cone and collision voltages, optimised to increase fragment ion intensity, were determined experimentally through manual tuning. This was achieved by infusing diluted PFAS standard solutions (10 ng mL⁻¹) at 10 μL min⁻¹ into the MS with combined LC flow of mobile phase. The determined MRM conditions for each analyte, including precursor ions (Q1), product ions (Q3), and optimised cone and collision energies (CE), are displayed in the Supplementary Information (Table S5).

**Table S3:**Ultra-high pressure liquid chromatography conditions

| **UHPLC Conditions** | | |
| --- | --- | --- |
| Analytical column | ACQUITY PREMIER HSS T3 Column (1.8 µm, 2.1 × 100 mm) | |
| Isolator Column | ACQUITY UPLC isolator column (2.1 mm X 50 mm) | |
| Mobile phase | A. Water: Methanol (95:5) (2mM ammonia acetate) | B. Methanol (2mM ammonia acetate) |
| Gradient conditions | Time (mins) | Mobile phase (B%) |
| A | 0.00 | 1 |
| B | 1.00 | 20 |
| C | 6.00 | 45 |
| D | 13.00 | 80 |
| E | 14.00 | 95 |
| F | 17.00 | 95 |
| G | 18.00 | 1 |
| H | 24.00 | 1 |
| Flow rate (ml/min) | 0.2ml/min | |
| Column temperature | 55°C | |
| Autosampler temperature | 10°C | |
| Injection volume | 7.5 µl | |
| Divert flow to waste | 0-4 and 17-24 min | |
| Injector switch valve | 17.00 and 17.20 min | |

**Table S4:** Mass Spectrometer conditions

| **MS conditions** | **Optimised conditions** |
| --- | --- |
| Ionisation mode | Negative electrospray ionisation |
| Capillary voltage (V) | 0.5 |
| Source temperature (°C) | 100 |
| Desolvation temperature (°C) | 350 |
| Desolvation gas flow (L/hr) | 900 |
| Cone gas flow (L/hr) | 100 |

**Table S5**: PFAS analyte and internal standard MRM transitions and parameters with corresponding internal standard pairings.

| **Target PFAS compounds** | | **Retention time** | **Quantitative MRM** | | | | **Qualifier MRM** | | | | **Internal standard** |
| --- | --- | --- | --- | --- | --- | --- | --- | --- | --- | --- | --- |
| Compounds | Monoisotopic mass (g/mol) | RT (minutes) | Q1  (*m/z*) | Q3 (*m/z*) | Cone (V) | CE  (eV) | Q1  (*m/z*) | Q3  (*m/z*) | Cone  (V) | CE  (eV) |  |
|  | | **Perfluorinated carboxylic acids** | | | | | | | | |  |
| PFBA | 213.996 | 5.30 | 212.9 | 169 | 14 | 10 | - | - | - | - | M4PFBA |
| PFPeA | 263.98 | 7.83 | 263 | 219 | 15 | 15 | - | - |  | - | M5PFPeA |
| PFHxA | 313.98 | 10.25 | 313 | 269 | 10 | 10 | 313 | 119 | 10 | 20 | M5PFHxA |
| PFHpA | 363.98 | 9.72 | 363 | 319 | 13 | 11 | 363 | 169 | 13 | 3 | M4PfHpA |
| PFOA | 413.97 | 12.26 | 412.9 | 369 | 14 | 9 | 412.9 | 169 | 14 | 9 | M8PFOA |
| PFNA | 463.97 | 13.21 | 463 | 419 | 15 | 10 | 463 | 219 | 15 | 15 | M9PFNA |
| PFDA | 513.97 | 14.00 | 513 | 469 | 15 | 10 | 513 | 219 | 15 | 10 | M2PFDA |
| PFUnDA | 513.97 | 14.69 | 563 | 519 | 10 | 10 | 563 | 269 | 10 | 15 | M7PFUnDA |
| PFDoDA | 613.96 | 15.26 | 613 | 569 | 15 | 13 | 613 | 169 | 15 | 20 | MPFDoDA |
| PFTrDA | 663.96 | 15.65 | 663 | 619 | 15 | 11 | 663 | 169 | 15 | 20 | M2PFTeDA |
| PFTeDA | 713.95 | 15.92 | 713 | 669 | 17 | 10 | 713 | 169 | 17 | 25 | M2PFTeDA |
|  | | **Perfluorinated sulphonic acids** | | | | | | | | |  |
| PFBS | 299.95 | 8.24 | 299 | 99 | 30 | 25 | 299 | 80 | 30 | 25 | M3PFBS |
| PFPeS | 349.95 | 9.91 | 349 | 99 | 30 | 28 | 349 | 80 | 30 | 28 | M3PFBS |
| PFHxS | 399.94 | 11.20 | 389.9 | 99 | 38 | 30 | 389.9 | 80 | 38 | 30 | M3PFHXS |
| PFHpS | 449.94 | 12.28 | 448.9 | 99 | 35 | 45 | 448.9 | 80 | 35 | 45 | M8PFOS |
| PFOS | 499.94 | 13.19 | 498.9 | 99 | 45 | 30 | 498.9 | 80 | 45 | 30 | M8PFOS |
| PFNS | 549.93 | 13.97 | 549 | 99 | 50 | 30 | 549 | 80 | 50 | 30 | M28:2FTS |
| PFDS | 599.93 | 14.65 | 599 | 99 | 60 | 30 | 599 | 80 | 60 | 30 | D5n-EtFOSAA |
|  | | **Perfluorinated sulphonamides** | | | | | | | | |  |
| FBSA | 298 | 9.89 | 298 | 78 | 50 | 20 | - | - | - | - | M3PFBS |
| FOSA | 498.95 | 14.77 | 498 | 83 | 45 | 25 | 498 | 78 | 45 | 25 | M8FOSA |
|  | | **Perfluorinated sulphonamidoacetic acids** | | | | | | | | |  |
| N-MeFOSAA | 570.97 | 14.36 | 570 | 419 | 30 | 15 | - | - | - | - | D3n-MEFOSAA |
| N-EtFOSAA | 584.99 | 14.73 | 584 | 526 | 25 | 15 | 584 | 419 | 25 | 20 | D5n-EtFOSAA |
|  | | **GenX** | | | | | | | | |  |
| HFPO-DA | 329.98 | 10.25 | 285 | 169 | 20 | 10 | 285 | 119 | 20 | 30 | M3HFPO-DA |
|  | | **Fluorotelomer sulphonates** | | | | | | | | |  |
| 4:2FTS | 327.98 | 9.54 | 327 | 307 | 30 | 15 | 327 | 81 | 30 | 20 | M2-4:2FTS |
| 6:2FTS | 427.98 | 12.19 | 427 | 407 | 30 | 17 | 427 | 81 | 30 | 30 | M2-6:2FTS |
| 8:2FTS | 527.98 | 13.99 | 527 | 507 | 35 | 20 | 527 | 81 | 35 | 20 | M2-8:2FTS |
|  | | **Perfluoroethers** | | | | | | | | |  |
| DONA | 377.98 | 11.32 | 377 | 251 | 10 | 10 | 377 | 85 | 10 | 25 | M4PfHpA |
| 9Cl-PF3ONS | 532.58 | 13.70 | 531 | 351 | 37 | 17 | 531 | 83 | 37 | 17 | M2-8:2FTS |
| 11Cl-PF3OUdS | 632.60 | 15.02 | 631 | 451 | 40 | 20 | 631 | 83 | 40 | 20 | M2-8:2FTS |
|  | | **Internal Standard** | | | | | | | | |  |
| M4PFBA | - | 5.30 | 217 | 172 | 14 | 10 | - | - | - | - | - |
| M5PFPeA | - | 7.83 | 268 | 223 | 15 | 15 | - | - | - | - | - |
| M3HFPO-DA | - | 10.25 | 287 | 169 | 20 | 10 | - | - | - | - | - |
| M3PFBS | - | 8.24 | 302 | 99 | 30 | 25 | - | - | - | - | - |
| M5PFHxA | - | 9.72 | 318 | 273 | 10 | 10 | - | - | - | - | - |
| M24:2FTS | - | 9.54 | 329 | 309 | 30 | 15 | - | - | - | - | - |
| M4PfHpA | - | 11.13 | 367 | 322 | 13 | 11 | - | - | - | - | - |
| M3PFHxS | - | 11.20 | 402 | 99 | 38 | 30 | - | - | - | - | - |
| M8PFOA | - | 12.26 | 421 | 376 | 14 | 9 | - | - | - | - | - |
| M2-6:2FTS | - | 12.19 | 429 | 409 | 30 | 17 | - | - | - | - | - |
| M9PFNA | - | 13.21 | 472 | 427 | 15 | 10 | - | - | - | - | - |
| M8FOSA | - | 14.77 | 506 | 78 | 45 | 25 | - | - | - | - | - |
| M8PFOS | - | 13.19 | 507 | 99 | 45 | 40 | - | - | - | - | - |
| M2PFDA | - | 14.00 | 519 | 474 | 15 | 10 | - | - | - | - | - |
| M2-8:2FTS | - | 13.99 | 529 | 509 | 35 | 20 | - | - | - | - | - |
| M7PFUnDA | - | 14.69 | 570 | 525 | 10 | 10 | - | - | - | - | - |
| D3-N-MEFOSAA | - | 14.36 | 573 | 419 | 30 | 15 | - | - | - | - | - |
| D5-N-EtFOSAA | - | 14.73 | 589 | 419 | 25 | 15 | - | - | - | - | - |
| M-PFDOA | - | 15.26 | 615 | 570 | 15 | 13 | - | - | - | - | - |
| M2-PFTeDA | - | 15.92 | 715 | 670 | 17 | 10 | - | - | - | - | - |
|  | | **Injection Standard** | | | | | | | | | |
| M4PFOA | - | 13.19 | 417 | 372 | 14 | 9 | - | - | - | - | - |

**PFAS Analytes and Limits of Detection (LOD) and Quantification (LOQ)**

The UHPLC-MS/MS method required 16 minutes for the complete elution of PFAS analytes with a total run time of 24 minutes. No endogenous peaks exceeding 20% of the LOQ response were observed, except for 6:2 FTS. Solvent blanks injected between samples throughout the method validation process showed no significant sample carry over effects.

**Table S6**: PFAS Analytes and Limits of Detection (LOD) and Quantification (LOQ)

| **PFAS analyte** | **LOD**  **ng/mL** | **LOQ**  **ng/mL** |
| --- | --- | --- |
| PFBS | 0.003 | 0.009 |
| PFPeS | 0.007 | 0.020 |
| PFHxS | 0.005 | 0.016 |
| PFHpS | 0.007 | 0.020 |
| PFOS linear | 0.008 | 0.024 |
| PFOS sum of isomers | 0.007 | 0.021 |
| PFNS | 0.004 | 0.012 |
| PFDS | 0.004 | 0.013 |
| PFBA | 0.013 | 0.039 |
| PFHxA | 0.019 | 0.039 |
| PFHpA | 0.012 | 0.036 |
| PFOA | 0.002 | 0.007 |
| PFNA | 0.005 | 0.016 |
| PFDA | 0.007 | 0.021 |
| PFUnDA | 0.002 | 0.005 |
| PFDOA | 0.002 | 0.007 |
| PFTrDA | 0.006 | 0.017 |
| PFTeDA | 0.012 | 0.035 |
| 4:2 FTS | 0.002 | 0.005 |
| 8:2 FTS | 0.001 | 0.004 |
| N-MeFOSAA | 0.013 | 0.040 |
| N-EtFOSAA | 0.001 | 0.004 |
| 9-Cl-PF3ONS | 0.001 | 0.002 |
| 11-Cl-PF3ONS | 0.002 | 0.005 |
| FOSA | 0.024 | 0.072 |
| HFPO-DA | 0.009 | 0.027 |
